# Facile preparation of the new organic ligands, schiff bases and metal complexes in well

**DOI:** 10.1186/s13065-025-01592-1

**Published:** 2025-08-04

**Authors:** Mervette El Batouti, El sayedH. El-Mossalamy, Jihad M. Aldesouky, Mohamed A. Khashaba, Howida A. Fetouh

**Affiliations:** 1 Chemistry Department, Faculty of Science, Alexandria University, Alexandria, Egypt; 2 Chemistry Department, Faculty of Science, Benha University, Banha, Egypt; 3 Clinical Pathology Department, Faculty of Medicine, Alexandria University, Alexandria, Egypt

**Keywords:** Antimicrobial, Schiff bases; metal, Ligand, Complex, Docking

## Abstract

For mitigating the wide spread antibiotic-resistant bacteria. This study aims: Simple synthesis of new series of coordination metal complexes: Cu(II), Co(II), Sm(III), Gd(III) and Tb(III) from the prepared Schiff base bis-hydrazones ligands I-VIII (derivatives of glyoxal, biacetyl and benzyl-hydroxybenzaldhyde and methoxysalicaldhyde). Structural features derived from elemental analysis (empirical formula), melting point (purity), nuclear magnetic resonance (^1^H, ^13^C) spectra and mass spectra. Vibrational IR spectra confirmed strong bonding between metal ions and ligands assumed the coordination sites are oxygen and nitrogen atoms of carbonyl C = O and azomethine CH = N groups. ^1^H-NMR spectra (chemical shift 3.5 ppm-10.388 ppm) confirmed all protons in the Schiff bases. Surface analysis SEM micrographs confirmed modified microstructure of 5^th^ ligand (LV) on complexation to Cu(II). Complex CuLV showed particle size range 276–367 nm. Optical activities of the metal complexes confirmed from electronic absorption spectra. Cu(II) complexes showed internal charge transfer bands. Powder X-ray diffraction pattern confirmed that CuLV complex formed in nm scale crystal with particle size range 13.91–35.49 nm. This complex is a potent antimicrobial agent in terms of the wide inhibition zone and low minimum inhibitory concentration (MIC) except for the fungi *A.Niger* and *C.Glabrata* (MIC 100 µgL^−1^ and 400 µgL^−1^ respectively).The promising inhibition of bacteria growth and low MIC suggested this metal complex as a new antibiotic. For its optimized geometry, molecular docking analysis predicted antibacterial activity and confirmed the observed weak antifungal activity corresponding to high MIC for *A.Niger* and *C. Glabrata fungal species*.

## Introduction

The research question formulated as: Can new coordination transition metal complexes rather traditional antibiotics easily prepared for evaluation as effective antimicrobial agents particularly against antibiotic-resistant microbes. Can antimicrobial activity confirmed experimentally and theoretically and can more biological activity be predicted. To combat antimicrobial resistant bacteria which resist conventional antibiotics, this innovative study involved preparation of novel metal complexes that can act as metallodrugs. The synergism of Cu(II) ion and ligand enhanced antimicrobial activity and disrupt biofilms or anti-resistant microbial structures. Generating reactive oxygen species (ROS), interfering with microbial DNA, or disrupting cell membranes which decreased bacteria resistance to the antibiotics (Abs). Cu complexes showed broad-spectrum efficacy against bacteria, fungi, protozoa and viruses [1].

Schiff bases compounds contain an azomethine imines group and their metal complexes are important in coordination chemistry [1]. Schiff bases are natural or synthetic compounds such as 4-{(E)-[4-(Cl-CH_3_) Ph] di-azenyl}-2-{[(4-substituted-Ph-NH]-CH_3_}. The bromo and porpoxy derivative are potent antibacterial and anticancer agent respectively as confirmed by molecular docking analysis [1]. Various Schiff bases easily prepared *via* condensing: amino carbonyl (aldehyde/ketone) compounds *via* sonication, microwave, acid catalyst and using ionic liquids. Schiff bases mono-, di-, or poly-dentate ligands-(transition, lanthanides and actinides) metal complexes prepared as anti (microbial, viral, cancer, diabetic and inflammatory) agents [2]. Anticancer Pt-Schiff bases complexes act by: binding and damage DNA, generating ROS, mitochondrial pathway, endoplasmic reticulum stress, inhibition epidermal growth factor receptor, activation immunogenic cell death inducing apoptosis [3].

Octahedral Cr(III), Fe(III), Mn(II), Co(II), Ni(II) and Cu(II)-(*E*)-*N*′-((2-OH-naphthalen-1-yl)methylene)-4-oxopiperidine-1-carbohydrazide complexes are optically active and antimicrobial agents. Docking simulation confirmed good binding score of ligands with bacterial adenylate kinase and peptide deformylase as well as fungal DNA polymerase enzymes [4]. Mn/Co/Ni/Cu-hydrazone Schiff base N‘(2OH-3-OCH_3_-benzylidene)-4-O-piperidine-1-carbohydrazide-Cl-di-aqua-nanocrystalline complexes are photoluminescence and antimicrobial agents. Thermal stability depends on the metal ion and followed the order: Ni(II) < Cu(II) < Mn(II) < Co(II) complexes [5].

There is a growing interest in synthesis of well-designed bis-hydrazones along with their metal complexes for applications as anti (cancer, viral and fungal) agents [6–8]. Our research continued on bis-hydrazones prepared from the reaction of glyoxal, biacetyl and benzyl with 2, 4-di-OH-bebzaldhyde and 4-OCH_3_-salicaldhyde. These compounds have great interest as potential compartmental ligands form mono- and bi-nuclear coordination complexes with various metal ions due to several donor centers in their molecular structure [9–11]. Coordination ability of the new prepared Schiff bases attracted our attention in elucidating structures of Cu(ll), Co(ll), Sm(lll), Gd(lll) and Tb(lll) complexes. Metal complexes of Schiff bases applied in design and synthesis of coordination complexes [10], conductors [11], host-guest chemistry [12], sensors [13], biologically active agents [9], heterogeneous catalysis and antibiotics [14–16].Their prompt widespread applications arose from ease synthesis method, staggering various and vast formed complexes [17]. Although several properties well known, novel application for such a peculiar class of molecules required further studies. Schiff bases-transition metal complexes contain tetra dentate ligands are electrochemically active [18]. The wide spread bacteria-resistant Abs and the low efficacy or prohibited side effects of these Abs made it necessary to prepare alternates new biologically active compounds of suitable molecular structure and mechanisms of action. Control over the molecular structure could improve the selectivity, pharmacokinetic and mitigating these side effects. The nm particle size Schiff bases metal complexes rarely reported. This study aims preparation, characterization of new Schiff bases and their corresponding metal complexes as well as exploring some of their promising biological activity.

## Materials and methods

Analytical grade solid chemicals (diketones (glyoxal, biacetyl or benzyl), hydrazine hydrate, phenyl hydrazine, di-OH-benzaldhyde, 4-methoxy salicaldhyde) purchased from Sigma Aldrich Co., purity 100%) used in this study as received without further purification or recrystallization. All the preparations followed the method reported elsewhere [6].

### Preparation of the organic ligands, schiff bases and metal complexes

**Fig. 1:**
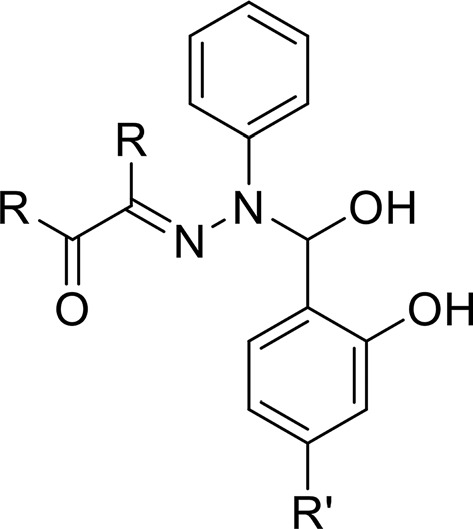
Chemical structure of ligands: (E)-3-(2-((4-R’-2-hydroxy-phenyl)-hydroxy-methyl)-2-phenyl-hydrazineylidene)alkan-2-on: L1(R = H, R’ (OH); LII (R = H, R’ (OCH_3_); LIII (R = CH_3_, R’ (OH); LIV (R = CH_3_, R’ (OCH_3_); LVI (R = Ph, R’ (OCH_3_); LVIII (R = Ph, R’ (OH)

**Fig. 2:**
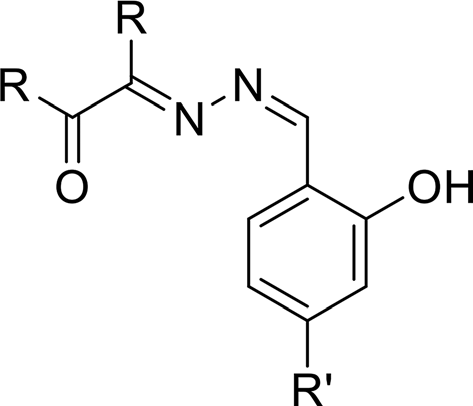
Chemical structure of ligands: (E)-3-(((Z)-4-R’-2-hydroxybenzylidene)-hydrazineylidene)-alkan-2-one). LV (R = Ph, R’= OH), LVII (R = Ph, R’ (OCH_3_)

0.1 mol diketone derivatives (glyoxal “1.5 mL”, biacetyl “2.8” g or benzyl"7.007 g”) dissolved in 25 mL ethanol in 100 mL flat-bottom flask. 0.1 mol hydrazine hydrate “3.2 mL” or phenyl hydrazine “6.56 mL” added dropwise to the previous solution in an ice bath. The resulting solutions refluxed for 5 h. On cooling the reaction mixtures, the products filtered, washed by ethanol and dried in vacuum desiccator over anhydrous CaCl_2_. Schiff bases of these ligands prepared as: A mixture: 2,4-di-OH-benzaldhyde “0.5 g” or 4-OCH_3_-salicaldhyde “0.5 g” and 0.1 mol each synthesized dihydrazide: 0.3 g: 2-hydrazonoacetaldehyde), 0.4 g:3-hydrazonobutan-2-on), 0.86 g 2-(hydrazono-1, 2-di-Ph-ethanon), 0.086 g (2-(2-Ph-hydrazono-acetaldehyde), 0.96 g (3-(2-Ph-hydrazono) butan-2-on) and 1.4 g (1, 2-di-Ph-2-(2-Ph-hydrazono)ethanon): 1:1 molar ratio in absolute alcohol contains few drops conc. HCl. Heat reflux conducted for 4 h provided the activation energy (Ea) for the slowest rate determining step in these multistep reactions. The reaction progress followed by TLC analysis until one spot formed. The reaction product separated on solvent evaporation and recrystallized from ethanol.Figures 1 and 2 summarized the molecular structure of the eight prepared bis-hydrazones ligand.

The purity confirmed by sharp melting point (m.p.). The chemical structures detected by CHN elemental analysis, IR and ^1^HNMR spectra. Solid chelates metal complexes of 1:1 M: L stoichiometric ratios prepared by mixing hydrated metal chlorides salts [CuCl_2_.2H_2_O, CoCl_2_.6H_2_O, TbCl_3_.6H_2_O, GdCl_3_.6H_2_O and SmCl_3_.6H_2_O] with a hot alcoholic ligand solution. Reaction mixture refluxed in a waterbath for 8–10 h, then left cooled to the room temperature. Solid chelates dried and preserved in a desiccator over anhydrous CaCl_2_ [6]. Molecular structure of ligands and metal complexes confirmed by infrared spectra (KBr) recorded using Perkin Elmer spectrophotometer1430 at frequency range 200–4000 cm^− 1^. Frequency reading calibrated using polystyrene film. ^1^HNMR spectra: recorded in CD_3_OH and (CD_3_)_2_SO respectively on Varian FT-200 MHz spectrometer. Scanning electron microscope JEOL- JSM5410-Japan used to determine microstructure of ligand or metal complex using sputter coated sample on gold thin film matrix [6]. Powder X-ray diffraction (pXRD) patterns recorded at 25 °C using Bruker D8 advance XR Germany diffractometer-copper anticathode target (Kα radiation illumination wavelength λ 1.54060 Å at 40 kV), Intensity data collected over 2θ range 5^o^-90^o^ by 0.02^o^ step, scanning rate 1^o^ min.^−1^. Intensity of reflected X-rays (arbitrary units) plotted versus incidence and reflection angles 2θ° [19]. Optical properties confirmed by UV-Vis. spectroscopy *via* recording electronic absorption spectra in the solution [19].

Antibacterial activity assayed in triplicates using well diffusion method, nutrient (NA) agar selective for bacteria and saboroud dextrose agar (Oxoid Lab., UK) for fungi. The diameter of the inhibition zone (IZ) around bacterial growth measured versus standard antibiotic (Ab). Sub-cultured tested organisms were: *S. pyogenes*, (*K.*,* S.) pneumoniae*, *P. mirabilis*, *E. fecalis*, *P. aeruginosa*,* E. coli* and *S.aureus*. The Abs *cephradine* and *cefepime* amphotericin B were used as positive control for bacteria and fungi respectively. Microbial cultures incubated at 37 °C for 24 h at 30 °C for 5 days [19]. The MIC of samples estimated in triplicates for each test organism in the nutrient broth. A lapful test organism (diluted to turbidity standard 0.5 McFarland) introduced into the tube. The broth media only seeded with test organisms (control). Tubes contained test organism’s bacteria and fungi cultures incubated at 37 °C for 24 h at 25–30 °C for 3–7 days. Turbid microbial growth monitored [20]. Molecular docking for CuLV complex to different amino acids-proteins sequence carried out using autoDock4 open-source software and protein data bank (PDB) code 10:4 CMQ, 2019.

## Results and discussion

The purity of the prepared ligands confirmed by the constant m.p. The elemental analysis confirmed empirical formula, Table 1 contained molecular data. The molecular weight range 286–452 g.mole^− 1^. The expected molecular structure and formula confirmed as the observed atomic percentages agree with the calculated values.

**Table 1:** Physicochemical properties and elemental CHN analysis data and tentative formula for ligands

Compound	Empirical Formula	Molecular weight	Microanalysis (observed versus calc.)				m.*p*.°C	Color
			C%	H%	*N*%	O%		
I	C_15_H_14_N_2_.O_4_	286	65 (64.9)	6.03 (4.895)	12.64 (9.79)	(22.3)	140–143	Brown
II	C_16_H_16_N_2_O_4_	300	67.61 (64)	5.92 (5.33)	12.48 (9.33)	(21.3)	110–112	
III	C_17_H_19_N_2_O_4_	314	66.75 (64.96)	6.76 (5.7)	10.17 (8.9)	(20.3)	120–123	Black
IV	C_18_H_21_N_2_O_4_	328	68.54 (65.85)	6.58 (6.097)	10.30 (8.53)	(19.5)	110–113	Brown
V	C_21_H_16_N_2_O_3_	344	71.66 (73.2)	5.31 (4.65)	7.00 (8.00)	(13.9)	130–133	Yellow
VI	C_28_H_25_N_2_O_4_	452	78.36 (74.34)	5.57 (5.309)	7.2 (8.06)	(14.1)	155–158	Green
VII	C_22_H_18_N_2_O_3_	358	70.76 (73.11)	5.07 (5.0)	6.33 (6.39)	(14.6)	170–173	Yellow
VIII	C_27_H_23_N_2_O_4_	438	75.43 (73.97)	5.53 (5.023)	6.33 (6.39)	(14.6)	120–122	Green

**Fig. 3:**
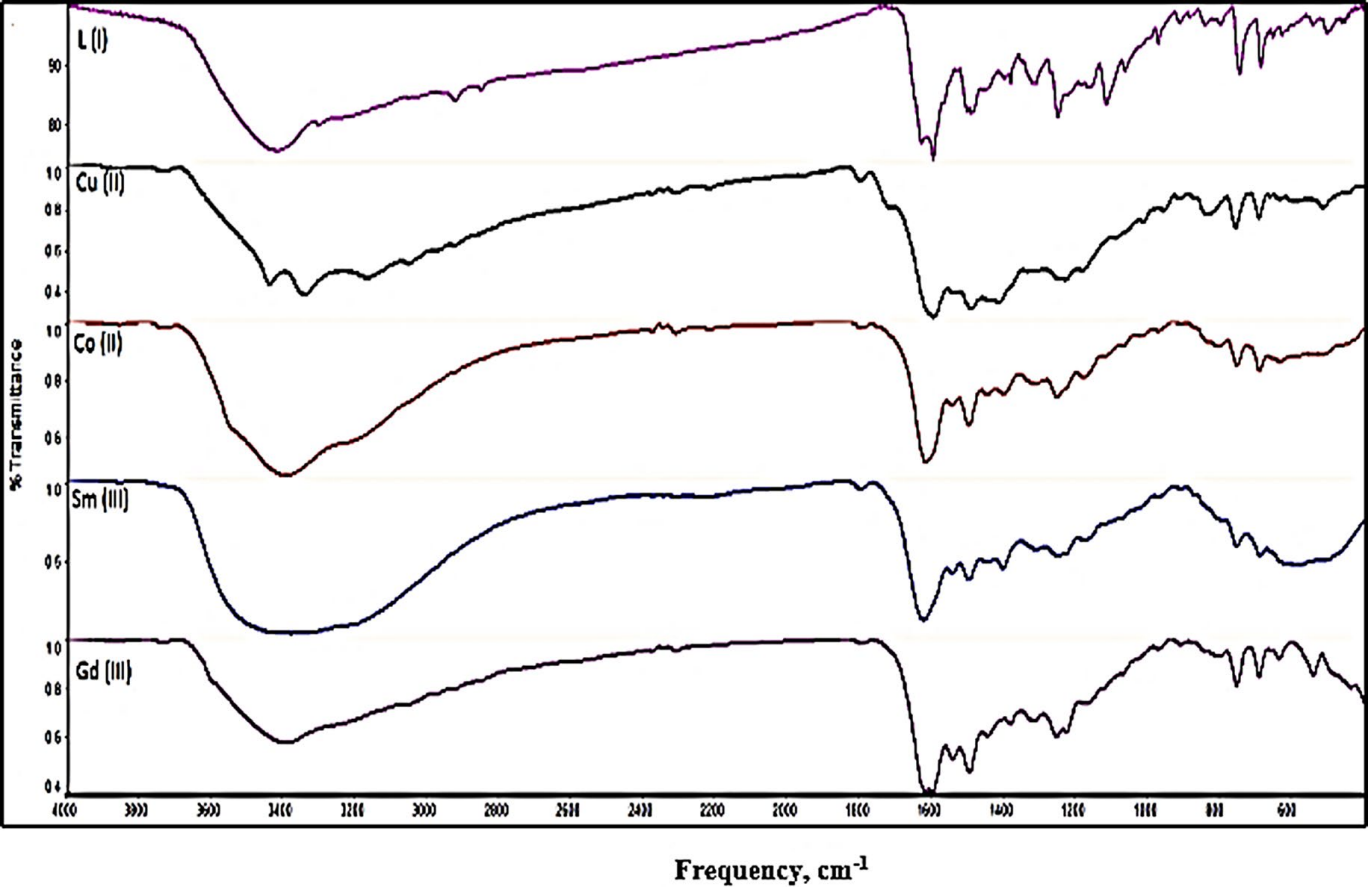
FTIR spectra, Ligand 1 and metal complexes

The representative FTIR spectra (Fig. 3) confirmed the bonding in the molecular structures of ligands and metal ions complexes [20]. The chemical structure of (Cu(II), Co(II), Sm(III) and Gd(III))-Schiff bases complexes deduced based on comparison the vibrational bands of chemical bonds to the characteristic vibration frequencies (cm^− 1^) listed in Table 2.

**Table 2:** Characteristic IR frequencies (cm^− 1^) of ligands and some metal complexes

Sample	OHυ	in plane δ OH	C = Oυ	C = *N*(say)υ	C = *N*(asy) υ	NH-Phυ	M-Oυ	M-Nυ
L_I_	3418	1385	1632	15,992	1253	1494	-	-
Cu L_I_	3345	1336	1598	1541	1233	1495	594	516
Co L_I_	3394	1406	1621	1546	1255	1449	693	637
Sm L_I_	3381	1405	1626	1547	1255	1449	692	586
Gd L_I_	3395	1448	1601	1545	1257	1498	543	440
L_II_	3425	1384	1600	1551	1254	1493	-	-
Cu L_II_	3336	1286	1594	1502	1222	1413	509	409
Co L_II_	3289	1403	1602	1510	1173	1457	624	582
Sm L_II_	3383	1283	1628	1509	1219	1458	693	634
Gd L_II_	3396	1278	1596	1507	1124	1458	543	643
L_III_	3417	1367	1632	1603	1239	1496	-	-
Cu L_III_	3357	1384	1604	1496	1230	1444	587	515
Tb L_III_	3381	1331	1652	1499	1241	1450	692	634
L_IV_	3418	1367	1602	1510	1240	1426	-	-
Cu L_IV_	3337	1280	1596	1505	1217	1417	590	511
Co L_IV_	3386	1278	1602	1508	1176	1454	698	585
Sm L_IV_	3384	1279	1657	1628	1240	1510	692	609
Gd L_IV_	3404	1428	1660	1597	1240	1508	544	440
L_V_	3424	1313	1679	1609	1219	1447	-	-
Cu L_V_	3447	1323	1674	1589	1217	1445	514	477
Co L_V_	3403	1319	1676	1612	1216	1447	460	440
Sm L_V_	3384	1319	1625	1507	1219	1445	689	648
L_VI_	3441	1340	1637	1603	1249	1511	-	-
Cu L_VI_	3340	1320	1667	1591	1211	1494	462	470
Tb L_VI_	3345	1350	1665	1590	1250	1500	695	590
L_VII_	3425	1305	1638	1599	1219	1550	-	-
Cu L_VII_	3332	1445	1677	1594	1218	1510	586	478
Tb L_VII_	3377	1304	1635	1600	1217	1515	645	485
L_VIII_	3424	1343	1603	1543	1248	1505	-	-
Cu L_VIII_	3346	1411	1671	1597	1212	1494	517	458
Tb L_VIII_	3363	1342	1639	1547	1251	1442	573	522

**Fig. 4:**
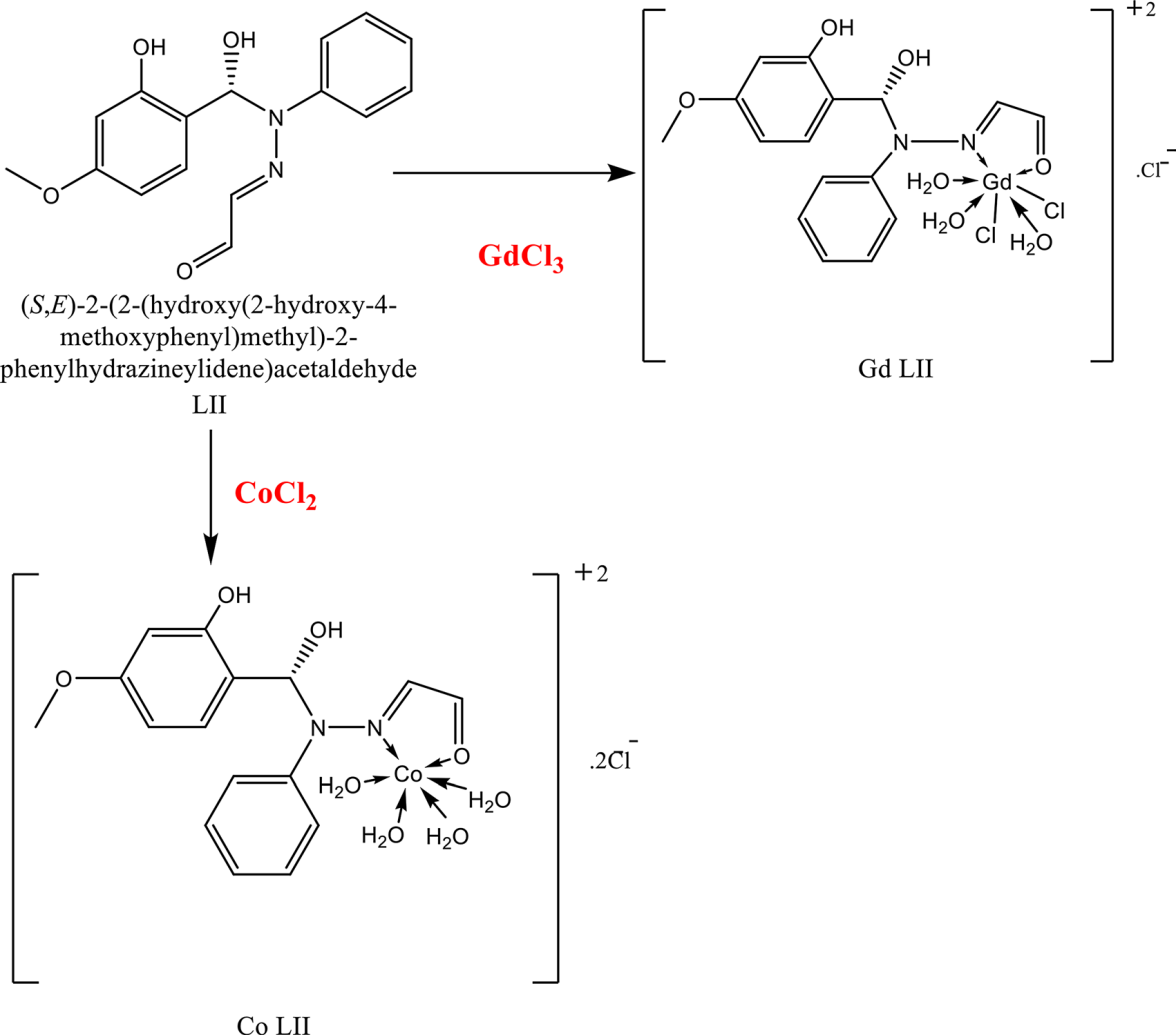
Bonding mode of ligand II with Co(II) and Gd(III) ions

**Fig. 5:**
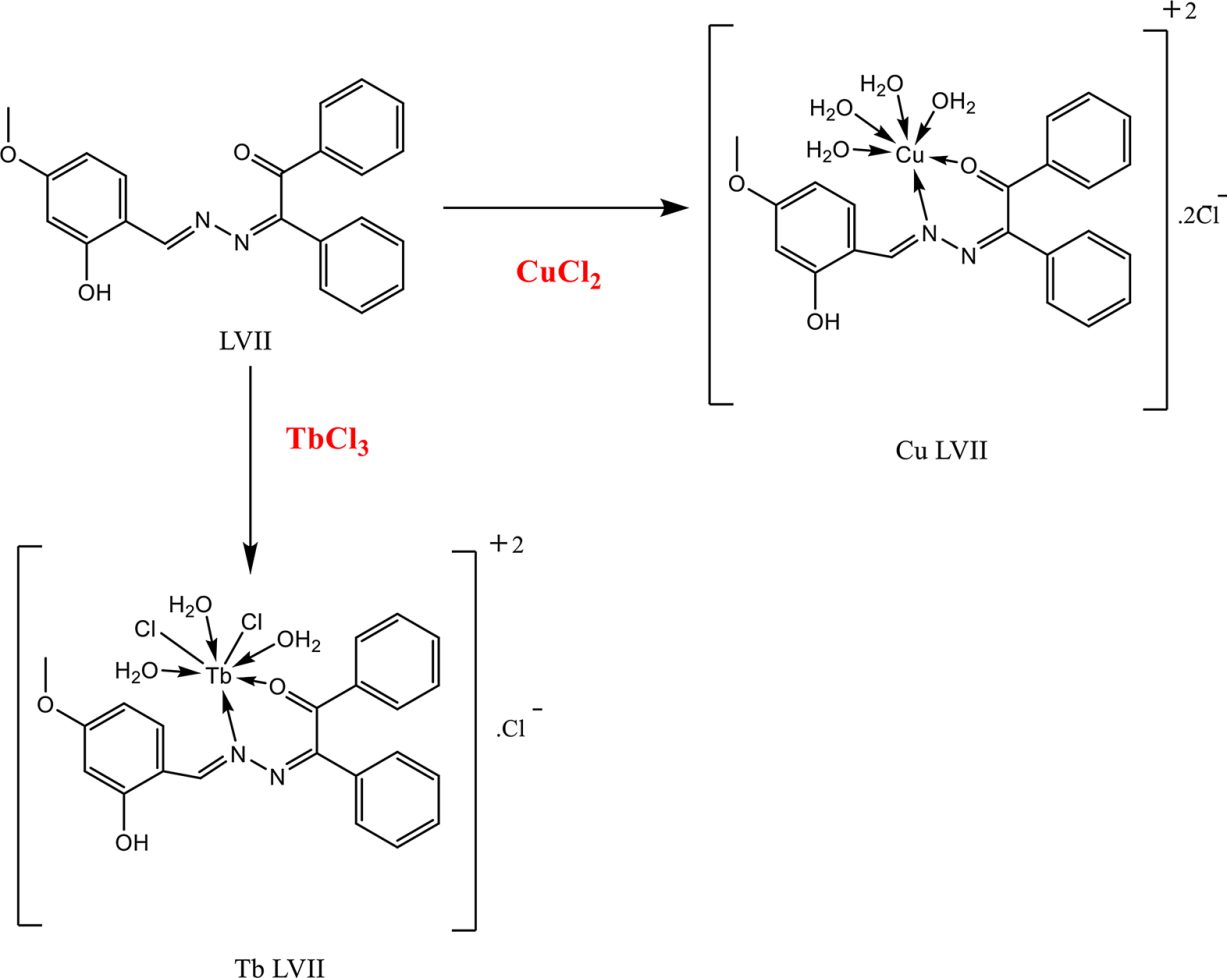
Bonding mode of LVII with Cu(II) and Tb(III) ions

On complexation, the broad band at frequency range 3345–3417 cm^− 1^ (stretch vibration of OH group) of coordinated water molecules appeared. Frequencies of coordinated functional groups (𝝊(C = N), 𝝊(C = O) affected by complexation with different degrees depending on strength of metal ion-π es interaction [21]. Medium strong bands at:1600–1678 cm^−1^, 1510–1608 cm^− 1^ in ligands (stretching C = O, C = N groups respectively) shifted to lower frequencies indicating binding the metal ions (M^N^). New bands at 460–698 cm^−1^ and 409–648 cm^−1^ assigned to M-O, N bonds respectively and indicated coordinate bond formation *via* bonding ketone C = O group, chloride ion, N-N = CH group of L to M^+n^. Bonding mode between metal ion and organic ketones ligand included coordinated water molecules represented in Figs. 4 and 5.

Metal ion binding ligand through N, O atoms. Cu(II) and Gd(III) ions showed six and seven coordination numbers respectively. Gd(III) ion chelated an extra chloride ion. The presence of the chloride ion in the outer sphere confirmed by the white AgCl precipitate determined on addition 0.1 M AgNO_3_ to 0.1 M complex solution (Volhard method) and the electrolyte (conductance around 5.5 × 10⁻⁸ Scm^− 1^). The prepared complexes are a coordination non-organometallic complex contain no carbon-metal bond. The ligands bind metal ion *via* N, O heteroatoms [6].

**Fig. 6:**
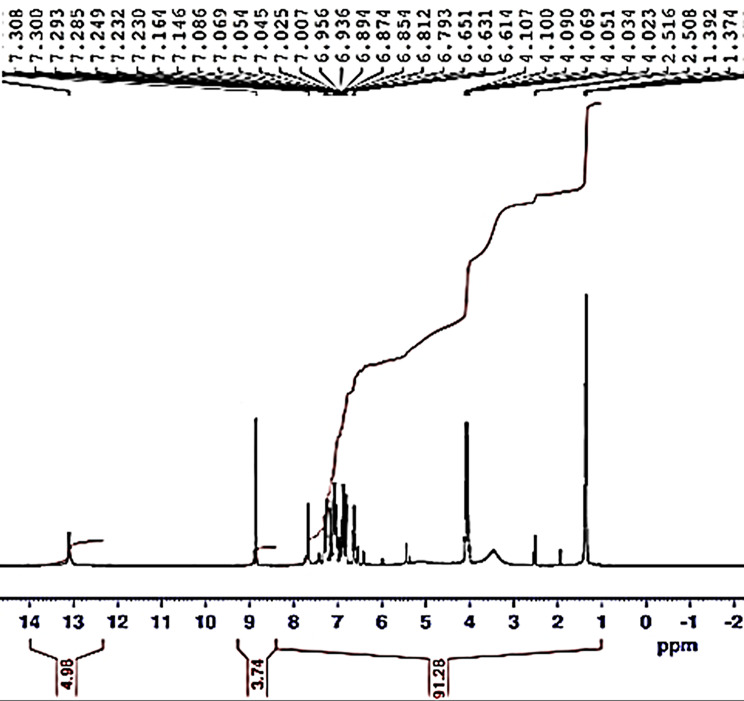
^1^HNMR spectra of Schiff base ligand V

Chemical shifts (δ; ppm) of the represented ^1^HNMR spectra [Fig. 6] recorded in Table 3. Protons (Hs) of azomethine CH = N group appeared as a singlet signal at the chemical shift (δ) range (8.053–9.771 ppm) in LI, II, V and VII. Signal at very high field side 3.5 ppm in ligands assigned as the phenolic Hs. Broadness attributed to hydrogen bonding (H.B.) interaction [17]. All the prepared ligands showed multiple signals in the range 7.1 ppm-7.5 ppm as aromatic Hs appeared at different positions due to different environments. In ligand I, II, signals at range (9.923ppm-10.388 ppm) aldehydic H (H-C = O). In ligand (IV-VII), signals at 3.8 ppm due to methoxy Hs (OCH_3_). Singlet signal (S, 3 H) in range 2.343 ppm-2.521 ppm in L III, LIV due to methyl Hs (CH_3_) [6].

**Table 3:** Chemical shifts (δ; ppm) for protons of ligands (I-VIII)

Ligand	(m,5 H)-Ar-H	(S,1 H) -OH	(S,3 H)-OCH_3_	(S,1 H)-CH = *N*	(S,1 H)-CH = O	(S,3 H)-CH_3_
I	7.175–7.456	10.9	-	8.053	9.923	-
II	7.200-7.432	10.4	-	9.771	10.388	-
III	7.188–7.368	10.1	-	-	-	3.5
IV	7.167–7.274	10.1	3.808	-	-	3.5
V	7.313–7.594	9.09	-	7.89	-	-
VI	7.189–7.532	10.1	3.847	-	-	-
VII	7.248–7.476	10.3	3.837	8.538	-	-
VIII	7.121–7.404	10.2	-	-	-	-

**Fig. 7:**
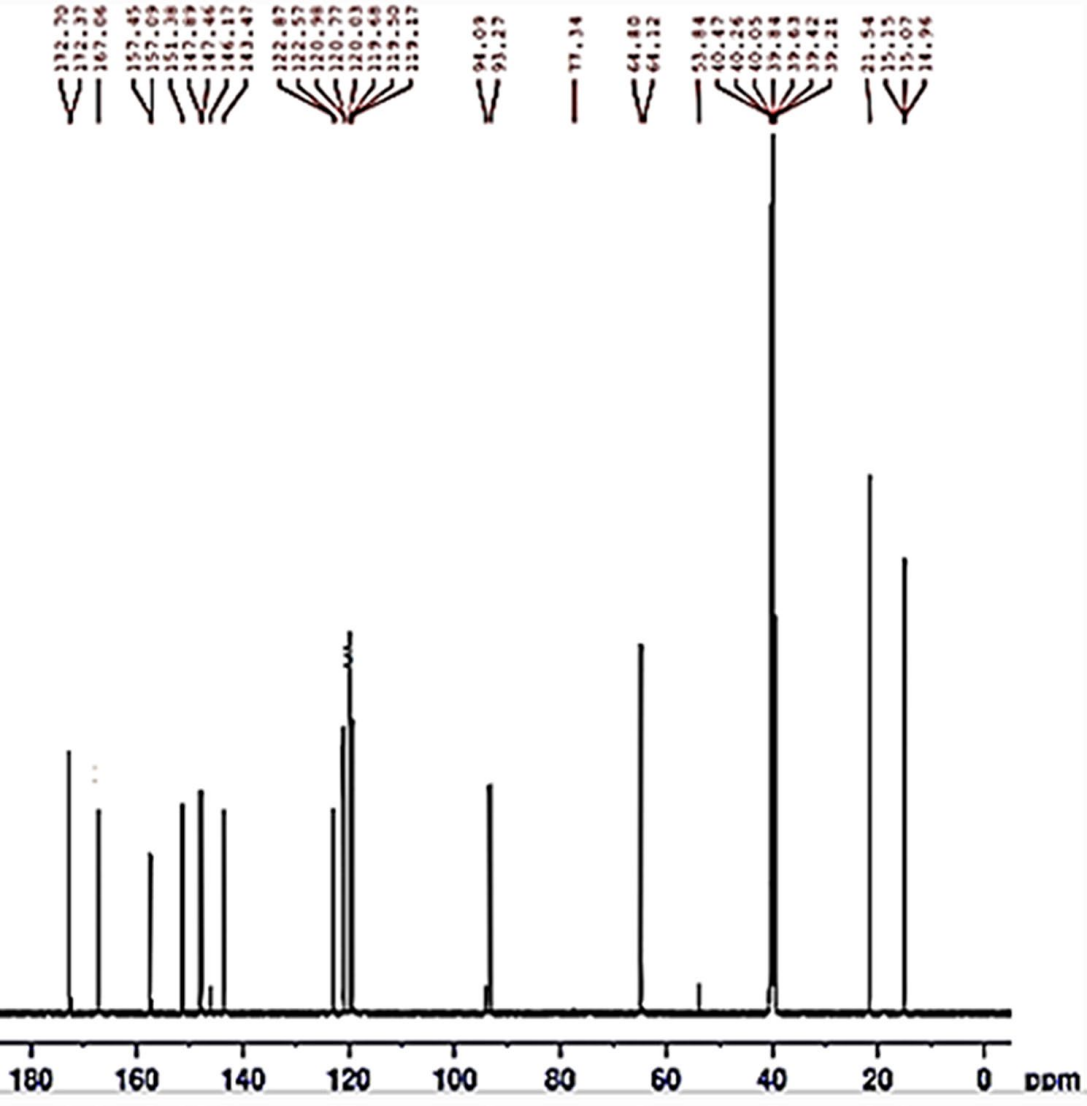
^13^C-NMR spectra of Schiff base ligand V

Numbers and types of carbon atoms (C) confirmed from ^13^C-NMR spectra, Fig. 7.

Signal at 40.8 ppm (DMSO), 15.25 (CO)ppm, 64.46 ppm CH, (128.36, 135.17, 121.92, 119.91, 119.45, 113.06, 111.28 ppm) aromatic C, 146.86 ppm phenolic C-OH, 143.15 ppm C-OR, 152.38 ppm (R’).

**Fig. 8:**
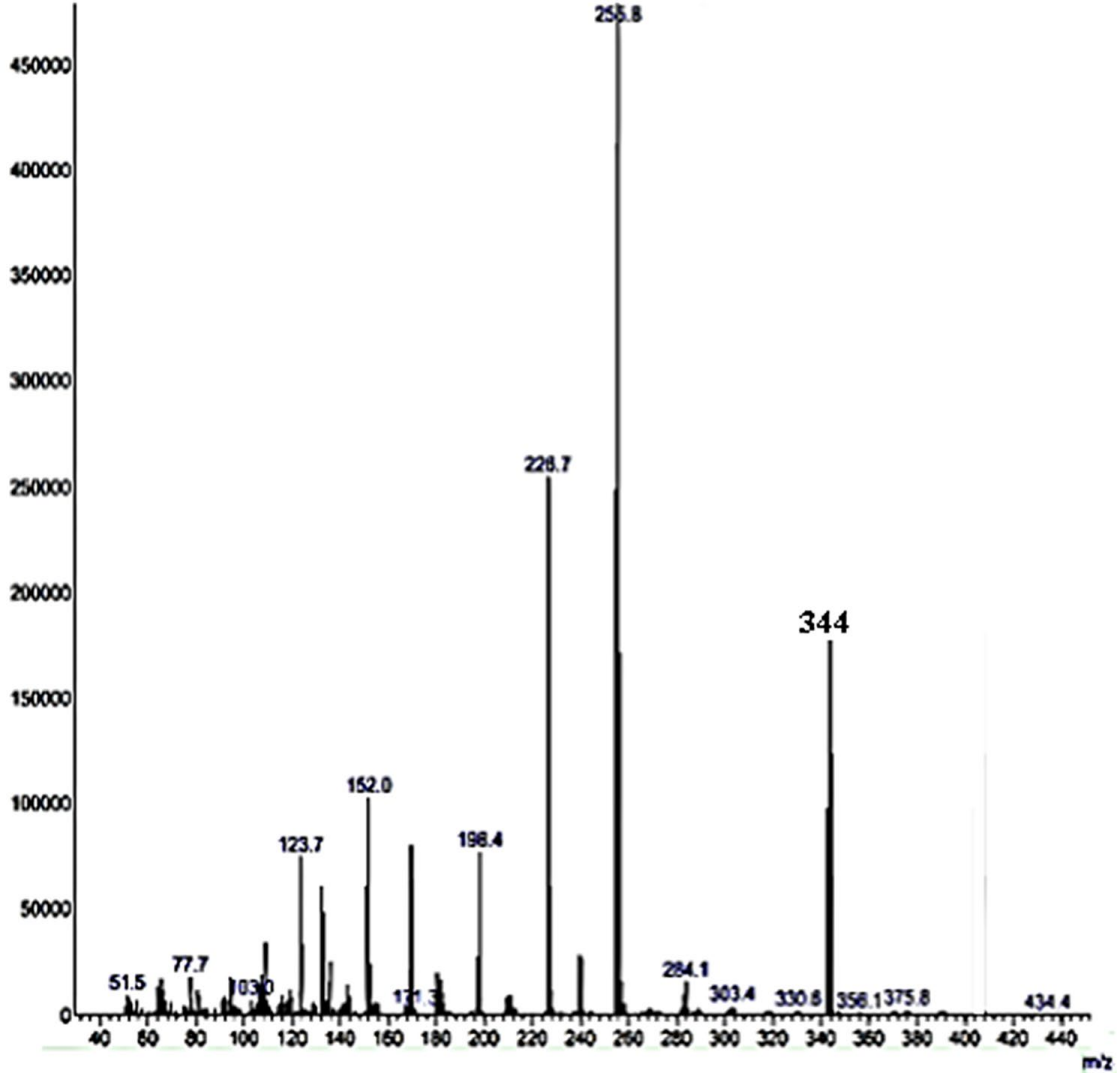
Mass spectra of Schiff base ligand V

The molecular weight confirmed from mass spectra [Fig. 8].

The last ion peak corresponding to the molecular weight (344 gmol^− 1^)of the ligand V.

**Fig. 9:**
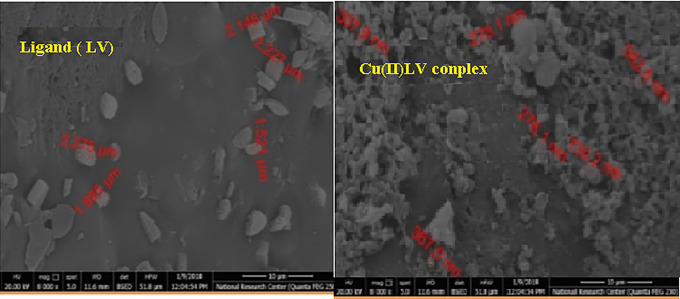
SEM micrograph for LV and Cu LV complex

Three dimensions SEM micrographs in Fig. 9 revealed scanned morphology of Schiff LV base and Cu(II)LV complex at 20 kV acceleration and 20000x magnification. Domain size of about 50 nm with void spaces observed in Cu(II)LV complex. Microstructure of Shiff base ligand changed from the micrometer scale: capsules, rods, crystalline separated shapes to reticulate, flower, cluster and polycrystals respectively [14]. Morphology of Schiff base remarkably changed to nm scale in Cu(II)LV complex.

The powder XRD pattern of Cu(II)LV showed good crystallinity with intense peaks especially at low theta values 13^o^, 17^o^ and 34^o^.

**Fig. 10:**
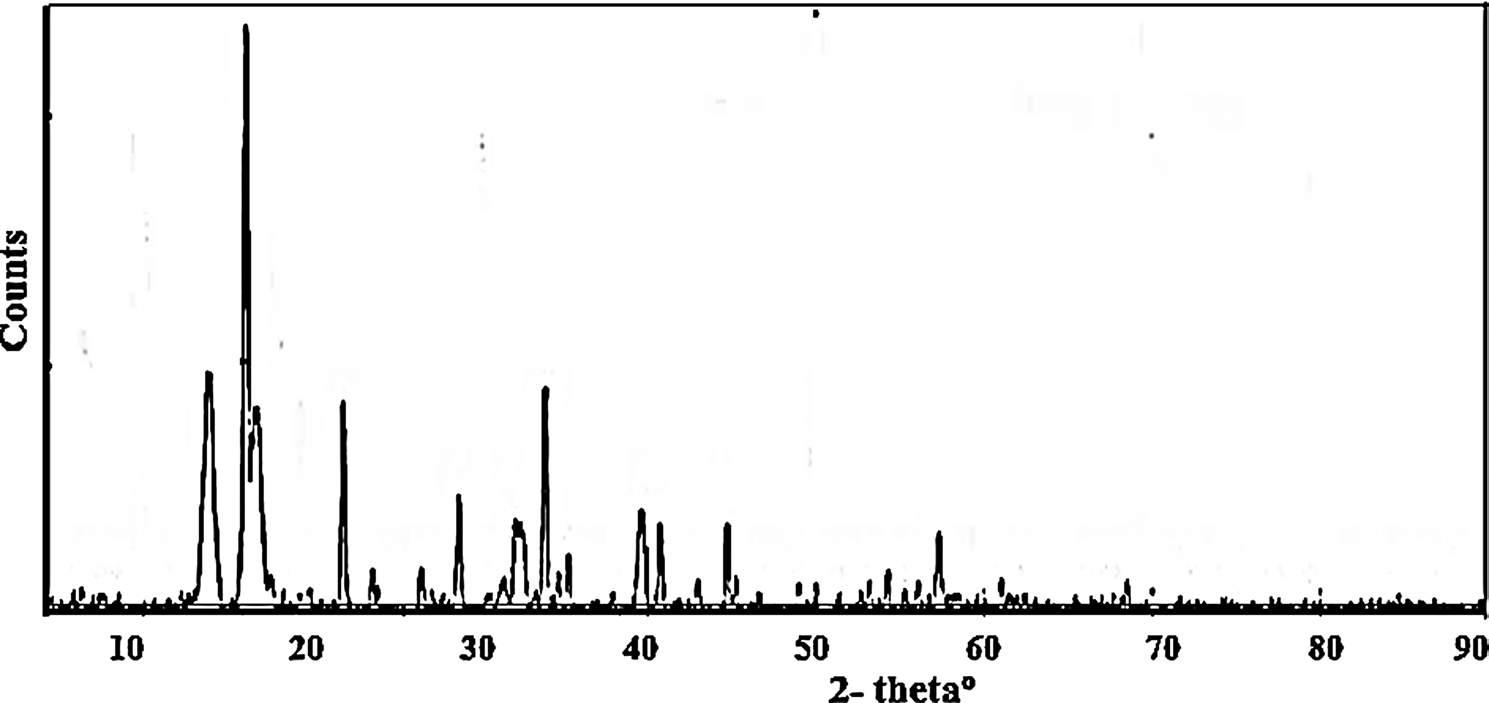
pXRD pattern of Cu LV complex. Broad diffraction patterns confirmed nm scale particles

The broad pXRD pattern confirmed nm scale particles and intermolecular hydrogen bonding: N-H···O and O-H···O) interaction [16].

Measuring interplanar d-spacing, integrated intensities, peak width& position followed Bragg’s law [22], Eq. 1:

1 $${\rm{n\lambda = 2d sin\theta }}$$

Where θ: incidence angles, d: inter planar distance between atomic crystal planes, λ: wavelength of the incident XR beam and n: is an integer plane order.

The crystal data obtained from comparing observed data with known standards 50,000 JCPDS pdF cards of related previously prepared inorganic materials in the crystallographic open data base (COD) [19]. The average grain particle size of nanoparticles estimated from diffraction intensity peak using Debye-Scherer Eq. 2 [23]:

2 $$T=C\lambda\:/\beta\:cos\theta\:$$

Where C: shape factor (~ 0.8–1.39), T: the mean size of the crystallite thickness and β: full-width at half-maximum (FWHM) of the peak (radians) after correction instrumental broadening.

Figure 10 demonstrated pXRD patterns of CuLV complex that confirmed nm scale particle size Diffraction peaks agreed with the reported standard data; The sharp observed characteristic Cu(II) ion peaks indicated sample purity [16]. The average grain particle size (T) in nm scale determined from the definite pXRD broad diffraction peaks, Table 4.

**Table 4:** Crystallite size of Cu LIII complex

Peaks No.	2θ^o^	Cos θ	D (°A)	β (FWHM) ^o^	T (nm)
17 13 34 42	16.728 13.915 28.473 33.868	0.9893 0.9926 0.9693 0.9566	5.296 6.359 3.132 2.645	0.603 0.630 0.427 0.244	13.91 13.26 20.05 35.49

The particle size ranged from 13.91 to 35.49 nm. The refine particle size attained at low 2-theta.

**Fig. 11:**
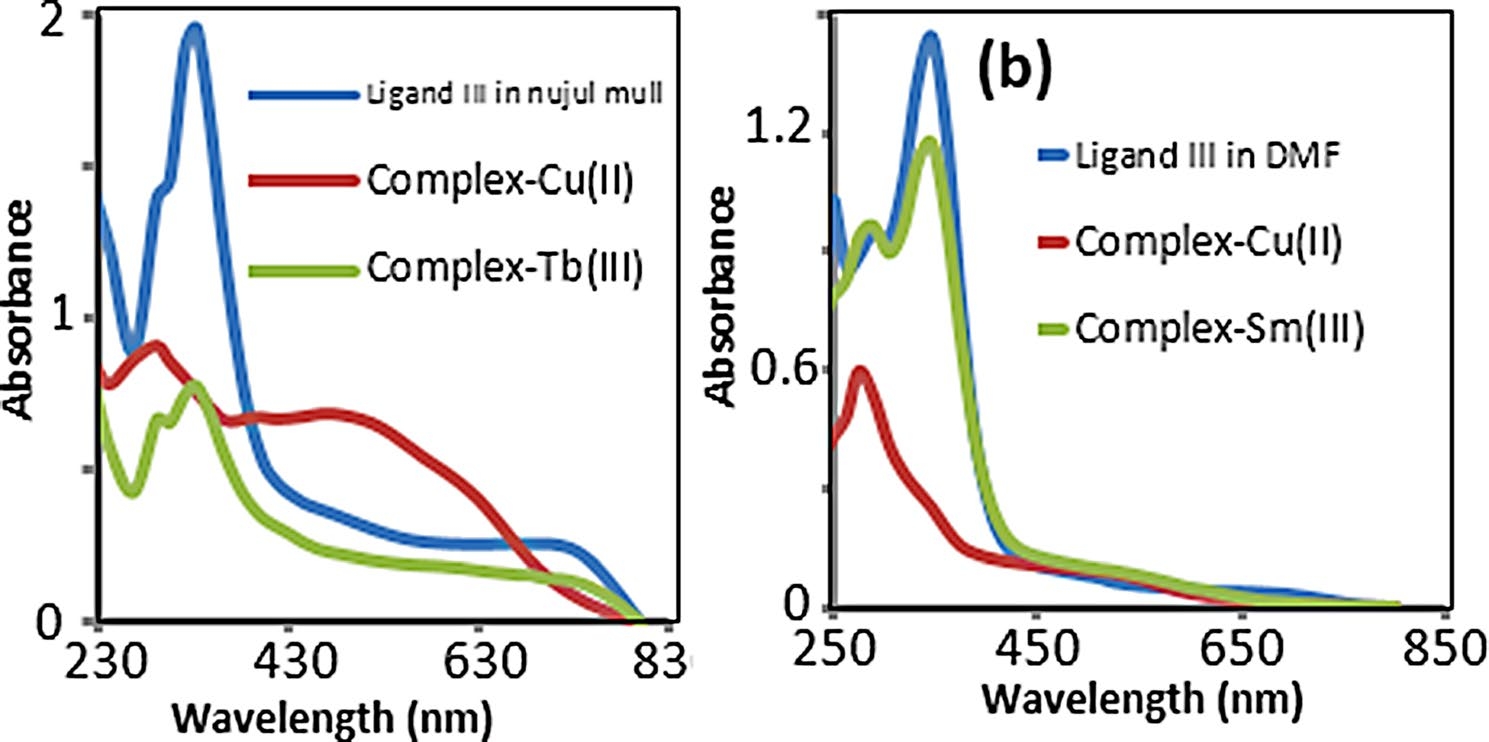
Electronic absorption spectra of ligand III and its metal chelate: **a**) Nujul mull and **b**) DMF

Electronic spectra of some metal complexes and the organic ligands represented in Fig. 11. Solid state Nujul mull electronic spectra of ligand showed more intense bands. Electronic spectral data listed in Table 5 [indicated an octahedral (Oh) geometry around all metal ions [21, 22]. Absorbance bands at 299–401 nm range assigned to intra-ligand CT transition (INCT). The effect of crystal field of the ligand represented in Table 5.

**Table 5:** Electronic spectral data (cm^− 1^) and related bonding parameters of culv complex

Complex	Electronic spectral bands		Energy levels	Covalent parameter
	Ligand V	Complex		
Cu L_V_	37,037 31,948 27,397	29,411 23,809 19,230	INCT ^2^E_2g_ → 2B1g, 2A1g → ^2^B_1g_	-

The unique electronic transitions and charge transfer (CT) in Cu(II) complexes attributed to d⁹ es configuration that affected the optical and the biological activity. The INCT due to the unique electronic transitions (localized es density) in the ligand framework, independent of metal coordination (2E₂g → 2B₁g and 2A₁g→2B₁g). INCT appear in both ligand and Cu complex but shift upon complexation due to crystal field. In Schiff base ligand, INCT bands occur in UV region (27397–37037 cm⁻¹). On complexation, these bands shifted to higher energies (19230–29411 cm⁻¹). Retained INCT bands in complex confirmed distorted Oh geometry and Cu-ligand interactions. The electronic transitions in Cu(II) complex displayed d-d transitions affected by the geometry of the metal complex. The transitions 2E₂g → 2B₁g (Jahn-Teller distorted Oh) and 2A₁g → 2B₁g (σ-bonding) reflected specific coordination environments. Zero covalency parameter confirmed the ionic character of Cu complex. The broad band at 439–630 assigned to ^2^E_g_ → ^2^T_2g_ (d-d*) transition in Oh crystal field in all complexes [19].

The optical activity of CuLV complex confirmed by electronic spectra. In perfect high spin Oh symmetry Cu(II)LV complex gave intense broad UV absorbance bands as the spin allowed vibrational and rotational transitions included during the electronic transition. Intense and multiple bands of CuLV complex as splitting d-energy levels gave axial distorted Oh symmetry around Cu(II) ion in crystal field under D_4h_ symmetry [24, 25].

**Fig. 12:**
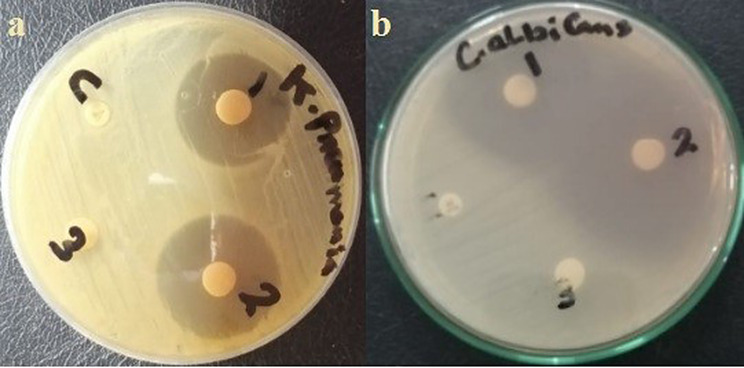
Inhibition zone: **a**) bacteria, **b**) fungi: (1) Positive control Ab, (2) Cu(II)LV complex, (3) LV, (4) DMSO

The antimicrobial activity for various pathogenic bacteria and fungi with known Robertson’s Cooked-Meat Broth (RCMB) shown in Table 6; Fig. 12.

**Table 6:** Inhibition zone for tested bacteria culv complex

Bacteria	S.pyogenes 010015	K.pneumonia 001009	*P*.mirabilis 010085	E.fecalis 010075	*P*.aeruginosa 010043	E.coli 010056	S.aureus 010027
IZ	21.7±0.48	22.5±0.48	19.6±0.48	18.2±0.53	21.5±0.54	22.3±0.34	22.4±0.48
MIC	0.88	0.88	2.9	14.63	1.85	0.88	0.88
Fungi	*A.niger* 2317	*A.flavus* 02426	*S.racemosum* 05922	*C.albicans* 05031	*C.glabrata* 05274	*F.oxysporum* 08213	
IZ	13.3±0.25	12.4±0.44	13.6±0.44	19.07	13.7±0.37	15.0±0.37	
MIC (µgL^− 1^)	100.10	15.02	16.26	15.30	400	2.9	

CuLV complex showed potent antibacterial and antifungal activity indicated by the large inhibition zones (IZ) [Fig. 12] and low MIC except for the fungi species *A.niger* and *C.glabrata* (MICs are 100 and 400 µgL^− 1^ respectively). It has both hydrophilic-hydrophobic character form H.B. interaction with bacteria ribosomal DNA: decreased protein, inhibited mitochondrial energy and bacteria growth.

**Fig. 13:**
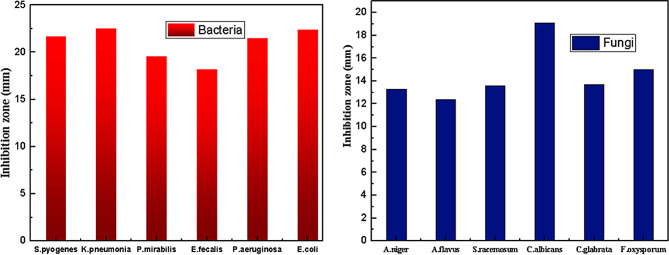
Inhibition zone of CuLV complex for various microbial species

The bio bar diagram shown in Fig. 13 showed IZ trend of Cu(II)LV complex towards various tested microorganisms.

The CuLV complex was more strongly antibacterial than antifungal. The strongest antifungal activity was against *C.albicans* (IZ 19.07 mm and MICs 15.3 µgL^− 1^). The complex CuLV showed wide inhibition zone (IZ) around bacteria culture. The wider IZ diffused into each other around *C.albican* fungi.

**Fig. 14:**
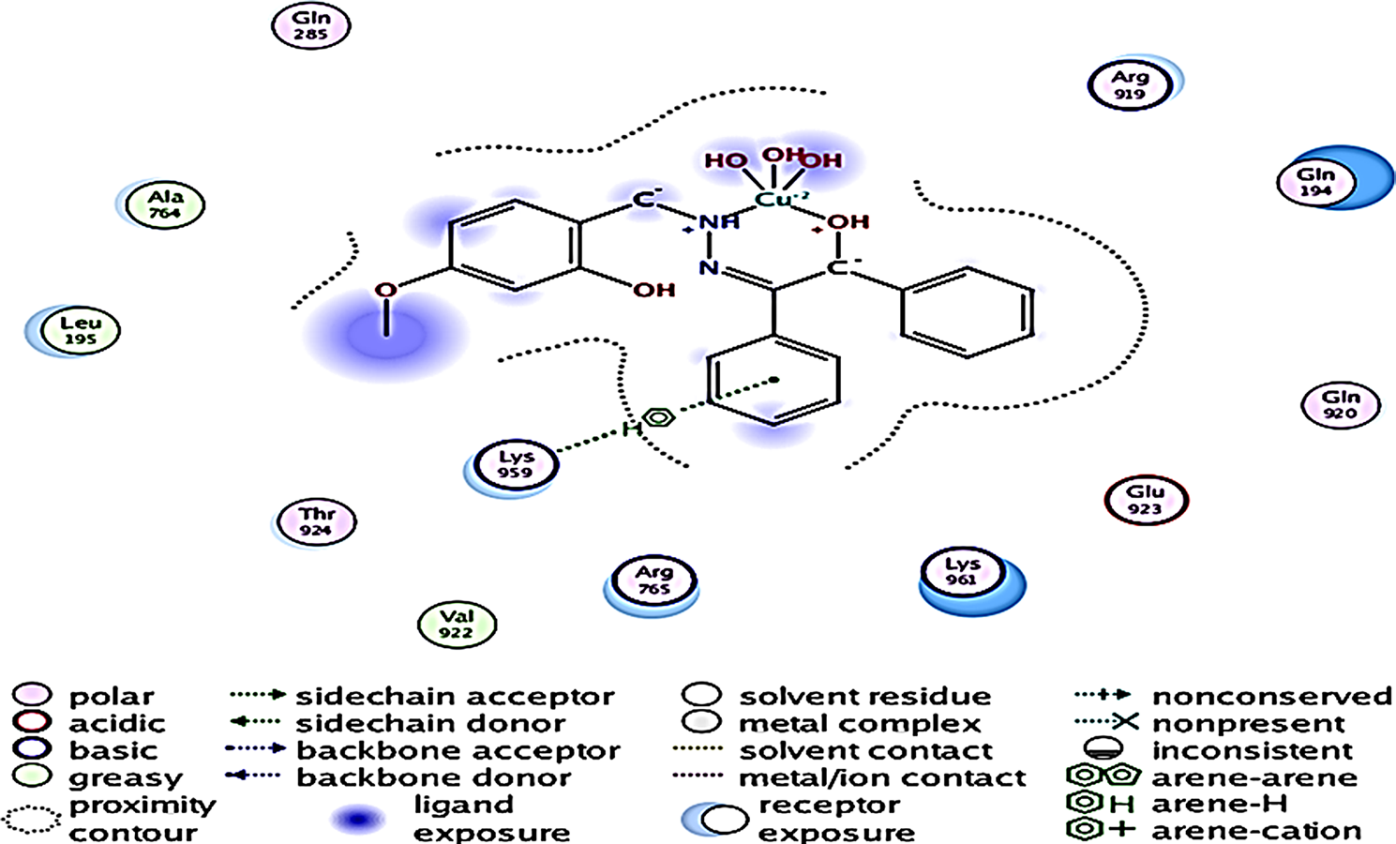
The simulated binding mode of CuLV complex with pyogene amino acids sequence

**Fig. 15:**
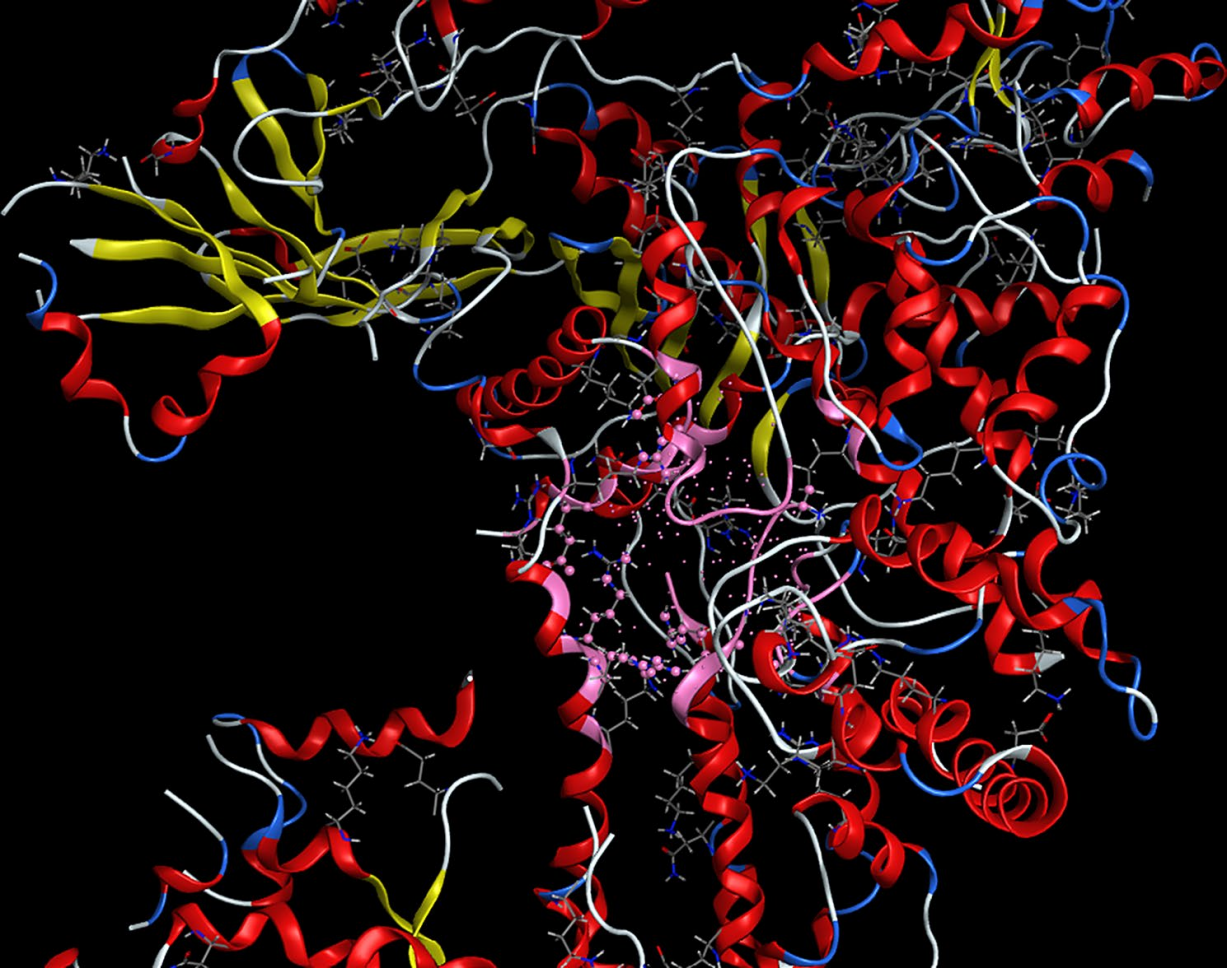
Molecular docking data for CuLV-*s.pyogenes* interaction: 2019.10 (PDB code 4 CMQ. The complex showed strongly affinity binding

**Fig. 16:**
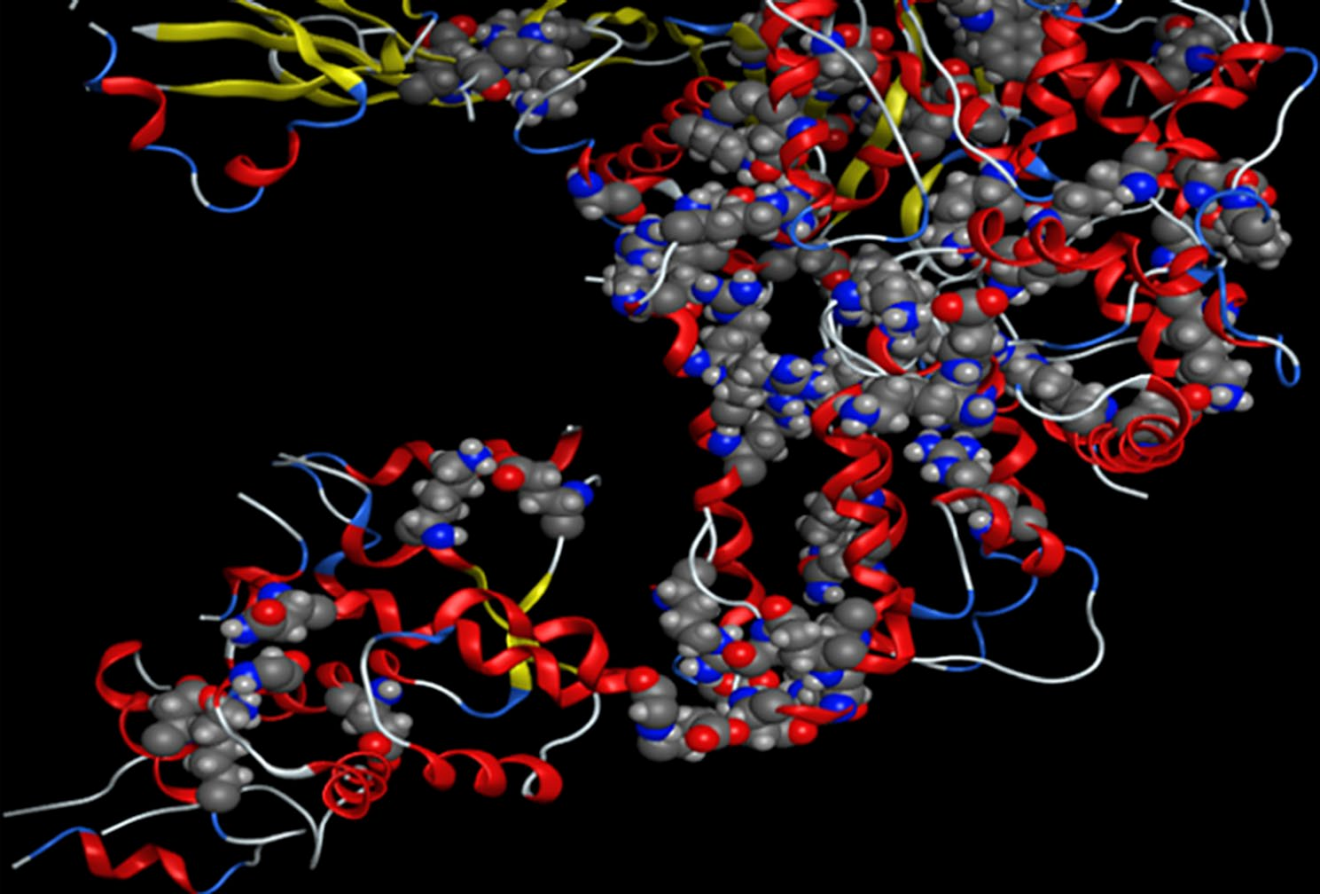
3D interaction: CuLV complex-active sites amino acid residues of *s.pyogene* (PDB 4 CMQ

**Fig. 17:**
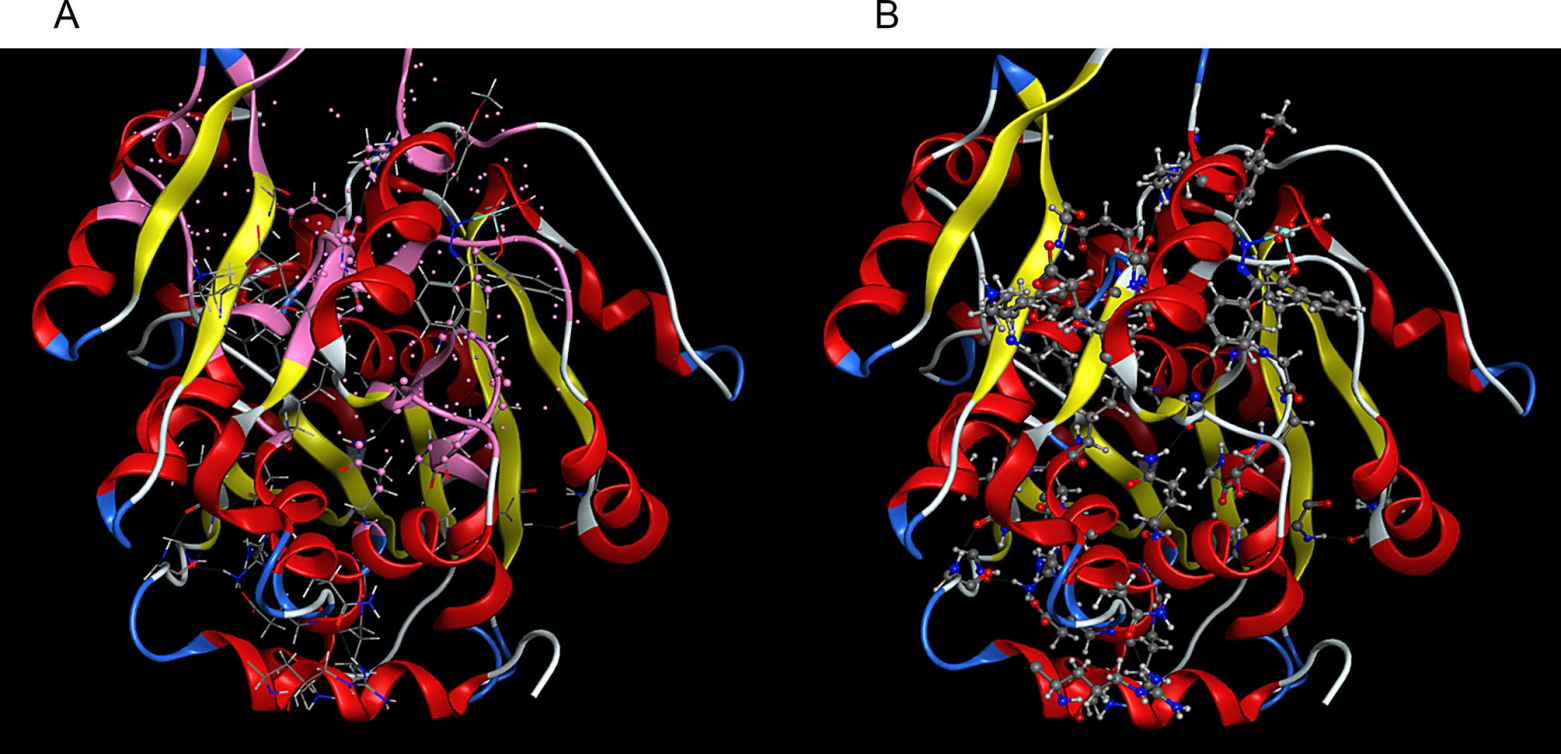
3D interaction of CuLV complex-active sites with amino acid residues of *S. Racmosum* protein

The 2D interactions of CuLV complex with active sites amino acid residues of pyogene (PDB code: 4CMQ) proteins shown in Figs. 14, 15, 16 and 17.

Molecular docking predicted preferred orientation of CuLV complex relative to binding protein that reflected molecular coupling strength or binding affinity (Van der Waals, electrostatics, H.B. and de-solvation. For example, mathematical scoring functions models (Table 7) confirmed protein-complex interactions.

**Table 7:** Representative molecular Docking results of ligand 6-ring CE

Receptor	Interaction	Distance	E (kcal mol^− 1^)	Binding energy score (S)
LYS 959 (A)	pi-H	3.49	-0.8	-5.8853

Negative binding energy indicated the stability of amino acid-CuLV complex cluster. Cu(II)LV complex is hydrogen and es donation to the bounded protein.

3D interaction of CuLV complex with the active sites on amino acid residues of *S.Racmosum* protein (PDB code: 1V71) shown in Fig. 17.

Docking CuLV complex with the same bacteria and fungi microorganisms experimentally examined did not give result with *C. Glabrata* and *A. Niger*. Results for the remaining tested microorganisms are so close and give good binding score (S). The strongest bonded *S.pyogenes*: -5.8853, the weakest bonded *K. pneumonia* − 4.7167. *F. oxysporum* (-5.5302) *via* 3 bonds reflecting more active sites and good binding energy [29, 30].

The performed molecular docking simulations data elucidated the binding energy (B.E) interactions between CuLV and target microbial proteins shown in Table 8. Binding affinity of CuLV complex confirmed through assessing the interaction. The complex acts as H/π-es donor interacted with LYS 959). Impossible docking for *C. glabrata* and *A. Niger* fungi confirmed high MICs and weak antifungal activity. In *F. oxysporum*:-5.5302 (4 bonded active sites). *K. pneumoniae*: lowest − 4.7167, the key interactions suggested as: hydro (philic/phobic) properties of Cu(II)LV complex facilitated H-B.with bacteria DNA ribosomes; disrupts bacterial DNA and inhibited both protein synthesis and ATP energy production from mitochondria. Subsequently inhibited bacteria growth.

**Table 8:** Molecular Docking data for culv complex against some target tested microorganism

Receptor (PDB code)	Microorganism	Interaction type	Distance (Å)	Energy (kcal mol^− 1^)	Binding score (S)	Binding interaction
*S. pyogenes (4CMQ)*	Bacteria	H.B. (GLY959) pi-cation (ASP235) pi-alkyl (LYS 959)	3.09 3.12 4.35	-0.8	-5.89	Strongest affinity H.B
*S. racmosum (1V71)*	Fungi	-	-	471.76	451.31	Weak
*K. pneumoniae*	Bacteria	pi-Cation (ARG 133)	4.44	-0.5	-3.65	Lowest

Positive binding for fungi species *S. racmosum* 1V71 indicated weak binding interaction with CuLV. The complex was less efficient antifungal against *Aspergillus Niger* and *C.glabrata* (MICs 100 and 400 µg/L) respectively.

**Fig. 18:**
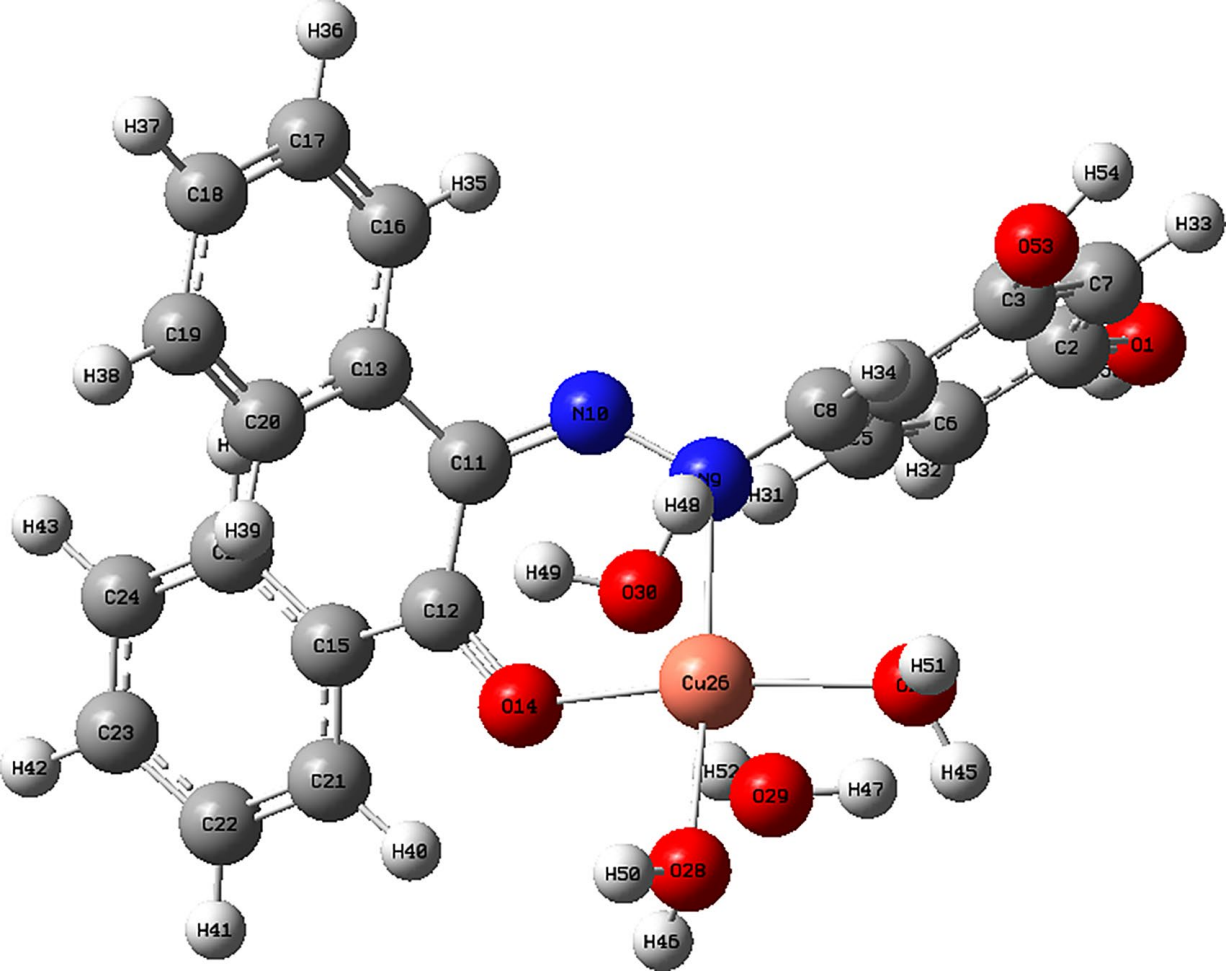
3D optimized structure of CuLV at DFT/B3LYP/GENECP

**Fig. 19:**
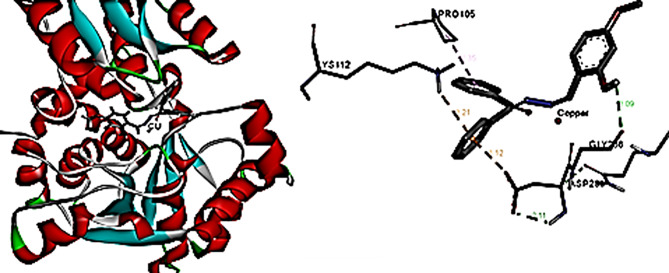
3D Binding Mode of CuLV with *S. pyogenes*4CMQ

**Fig. 20:**
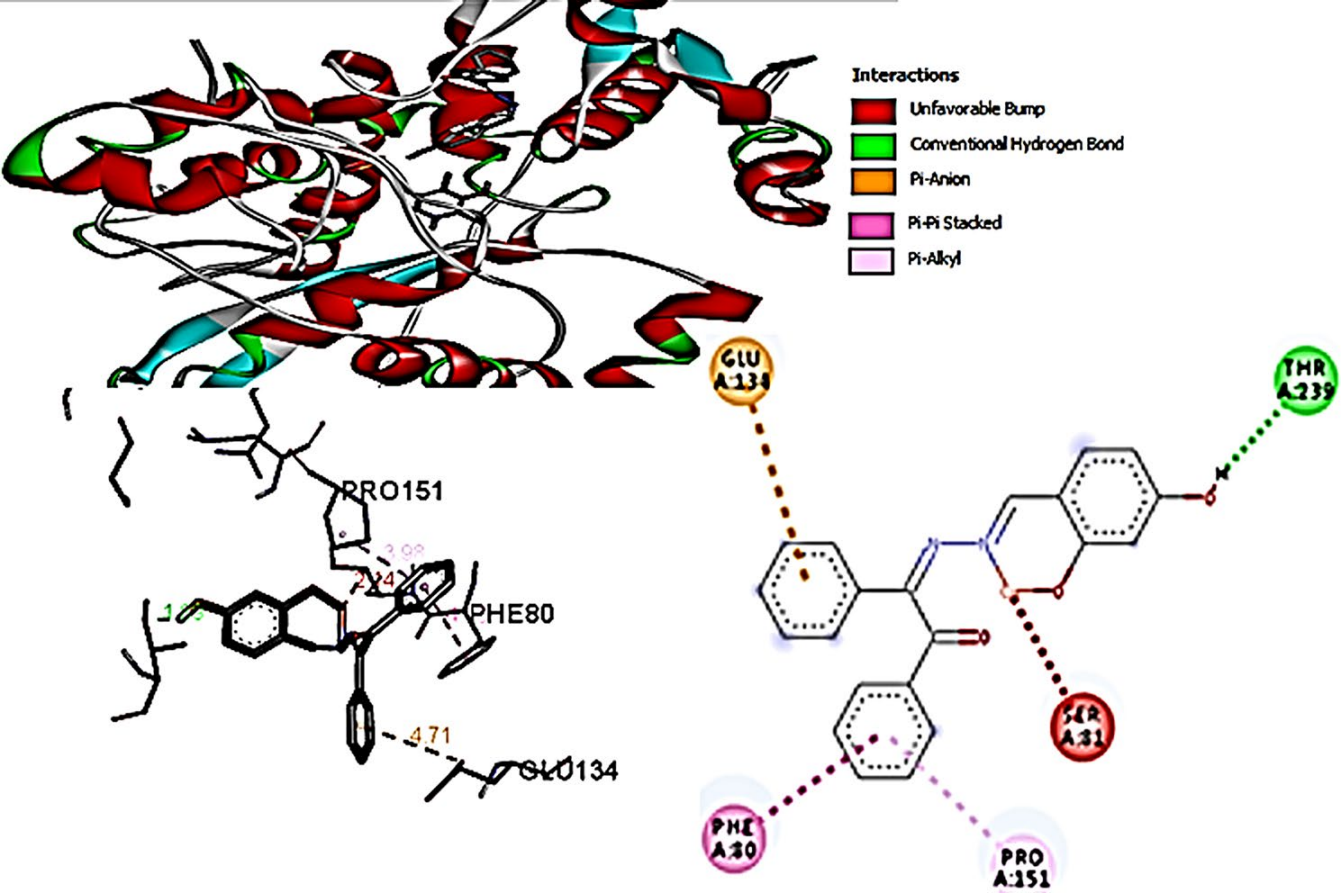
3D Binding Mode of CuLV with *Fusarium oxysporum* 4CMQ

Complex showed a high binding affinity for *Streptococcus pyogenes* (PDB 4CMQ). The preferential orientation stabilized by H.B. and es donor-acceptor interactions. Binding Mode: Figures 17, 18, 19 and 20 showed CuLV complex formed a conventional H.B. with GLY 959 (Distance: 3.09 Å), π es-Cu(II) interaction with ASP 235 (3.12 Å) and π-alkyl interaction with PRO105 (3.12 Å) in active site of *S. pyogenes*: binding energy − 0.8 kcal mol^− 1^ and docking score − 5.9 (Table 7). Negative sign confirmed thermodynamically stable CuLV-protein complex.

**Fig. 21:**
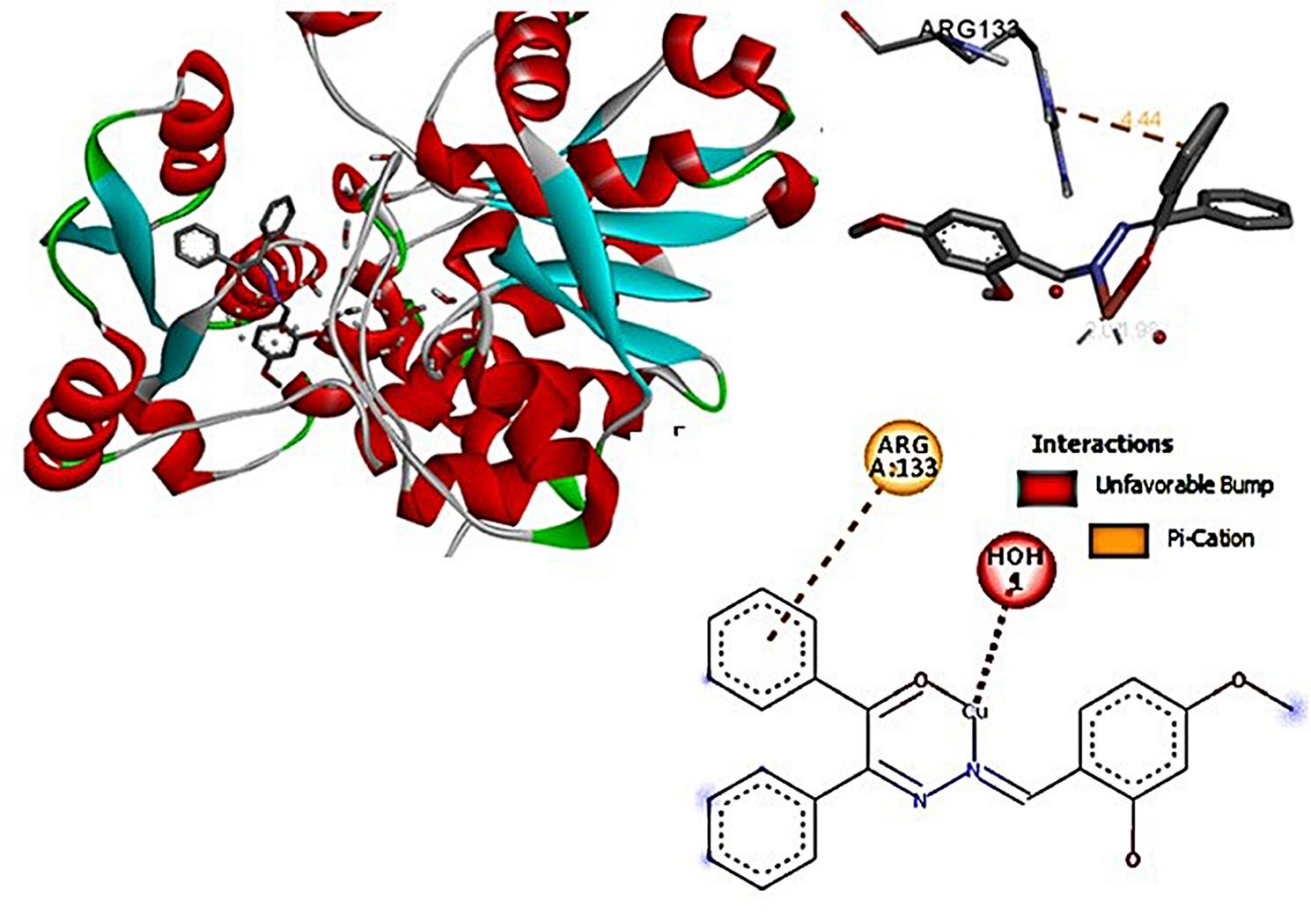
3D Binding mode of CuLV with *K. pneumoniae* 4CMQ

Molecular dynamics (MD) simulations confirmed stability of CuLV complex at protein binding site over 10ns-100 ns simulation time, the dynamic behavior of bonded protein confirmed the complex remained stable undissociated with retained initial conformation. 3D Interactions: Figures 18, 19, 20 and 21 highlight 3D conformations binded *S.pyogenes* and *S.racmosum*. Multi-binding sites shown in *Fusarium oxysporum* (-5.5302), *Klebsiella pneumoniae* displayed the lowest affinity − 4.7167. Impossible docking for *C.Glabrata or A.Niger* confirmed high MICs. Molecular docking validates its strong affinity for *S. pyogenes* and other susceptible strains supported by favorable B.E. and multi-sites interactions. The promising score results suggested CuLV recommended for developing antimicrobial agent.

In the thermally stable electrically conductive Oh metal complexes: Ni(II) and Cu(II) ions-2-[‘2-(4,5-dimethyl thiazolyl)azo]-imidazole:2-NH_2_-4,5-di-CH_3_-thiazo-imidazole, the binding. coordination centers were: (N, N, N), C = N (thiazole), azo N = N, C = N imidazole moiety. Molecular docking confirmed hydrophobic and H.B. to protein receptors. Ligand and metals complexes are moderate antimicrobial and anticancer agents [26]. Active complex: (E)-1-(4-((2,4-di-OH-6-CH_3_-Ph) diazenyl)Ph)ethanon-Ag(I) suggested as antibiotic, antioxidant strong bonded (PDB 3T88) of *E.coli* protein − 6.7818,   -6.7928 kcal mol^− 1^ respectively. Ag(I) on active site of cervical cancer receptor (4XR8) demonstrated higher B.E. -6.2631  kcal mol^− 1^ than ligand − 5.8561  kcal mol^− 1^ [27]. For tridentate ligand:2-[2′-(6-OCH_3_-benzothiazolyl)azo]-5-di-CH_3_-NH_2_-benzoic acid-Pt(IV)/Au(III) complexes, molecular docking confirmed receptor strong binding protein kinase 1HK7 and potent cancer-cell antiproliferative activity [28]. Molecular docking for Pd(II)/Co(III)/Pt(IV)-2-[2′-(benzimidazole)azo]-4,6-di-CH_3_ benzoic acid complexes showed B.E.above 5 kcal mol^− 1^ and RMSD 1.41 2.01 Å suggested molecular targeting [29].The antimicrobial activity of azo dye ligands and Ag(I), Pt(IV) and Au(III) complexes confirmed from docking crystal structure to FGF Receptor 2 kinase in cancer cells [30]. PdII/IV-[2′-(3,4-*di-*CH_3_-COOPh)*azo*]-5-CH_3_ imidazole complexes interacted with BCL2 and BAX BH3 peptide-RCSB protein [31]. For benzodiazepines, antibacterial activity confirmed from docking simulation *via* inhibition androgen receptor prostate cancer mutant H874Y ligand binding domain bounded testosterone and AR 20–30 peptides 2Q7K. Docking parameters confirmed potent anticancer activity against human prostate cancer [32]. Multidisciplinary biological activity confirmed by strong interaction with EGFR proteins [33].

Metal complexes hydrazone Schiff base N‘(2OH-3-OCH_3_-ybenzylidene)-4-oxopiperidine-1-carbohydrazide (H_2_L): [M(HL)(Cl)(H_2_O)_2_], M = Mn, Co, Ni, Cu are optically active (absorbance at λ334 nm, fluorescence emission at λ527–545 nm) and biologically active against various microbial species [34]. Cr(III)/Mn(II)/Fe(III)/Co(II)/Ni(II)/Cu(II) and Zn(II) (1:1 stoichiometry (M: L)-hydrazone Schiff base ligand, *N*’-(4-(di-ethyl-NH_2_-2-OH-benzylidene)-4-O-peridine-1-carbohydrazide complexes: are non-electrolytes, covalently π-bonded with axial distorted structure. Photoluminescence caused by ligands and M^+ n^. Thermal stability and the antimicrobial activity enhanced by complexation and varied with M^+ n^ nature [35]. Cr, Mn, Fe, Co, Ni, Cu complexes of Schiff base are more potent antibacterial than the parent hydrazone ligand [36]. The ligand and metal complexes added a significant contribution to the multidisciplinary applications of coordination metal complexes [5, 26, 37–40].

## Conclusion

New Schiff bases ligands (Ls) (I-VIII) derived from glyoxal, biacetyl and benzyl prepared. FTIR spectra confirmed these ligands showed many coordination sites to several metal ions through carbonyl C = O oxygen of the ring and nitrogen of the azomethine CH = N group. All complexes had Oh geometry. The structural features of the new series Schiff bases derived from elemental analysis, m.p. Vibrational IR bands (confirmed strong bonding to the metal ions.^1^HNMR spectra at chemical shift range (3.5 ppm-10.388) confirmed all protons. SEM micrographs confirmed microstructures. Complexes showed good optical activities. Cu(II) complexes showed internal charge transfer bands and nm scale semi crystals. The particle size range 13.91–35.49 nm confirmed from pXRD diffraction pattern. CuLV complex showed potent antimicrobial activities with low MIC for many bacteria and fungi species as evidenced by large inhibition zones and small MIC against many microbial species except *Aspergillus Niger* and *Candida glabrata* strains. The low efficacy expected from the high MICs 100 µg/L and 400 µg/L respectively. Molecular docking of the complex confirmed the experimental results: The potent broad-spectrum antimicrobial activity except the two fungi *Aspergillus Niger* and *Candida glabrata.*

## Data Availability

“Data is provided within the manuscript”.
